# Thromboembolic and hemorrhagic risks after vaccination against SARS-CoV-2: a systematic review and meta-analysis of randomized controlled trials

**DOI:** 10.1186/s12959-021-00340-4

**Published:** 2021-11-13

**Authors:** Noppacharn Uaprasert, Krissana Panrong, Ponlapat Rojnuckarin, Thita Chiasakul

**Affiliations:** 1 Division of Hematology, Department of Medicine, Faculty of Medicine, Chulalongkorn University and King Chulalongkorn Memorial Hospital, Thai Red Cross Society, Bangkok, Thailand; 2 Research Unit in Translational Hematology, Faculty of Medicine, Chulalongkorn University and King Chulalongkorn Memorial Hospital, Thai Red Cross Society, Bangkok, Thailand

**Keywords:** Thromboembolism, Hemorrhage, Vaccine, SARS-CoV-2

## Abstract

**Background:**

Thromboembolic and bleeding events after vaccination against severe acute respiratory syndrome coronavirus 2 (SARS-CoV-2) are major public concerns leading to vaccine hesitancy. Due to low incidence, an individual randomized controlled trial (RCT) is underpowered to determine whether SARS-CoV-2 vaccines increase the risks of thromboembolism and hemorrhage.

**Methods:**

We performed a literature search using PubMed, EMBASE, Cochrane, medRxiv databases, and reference lists of relevant articles to identify RCTs that reported thromboembolic, hemorrhagic events, and thromboembolism/hemorrhage-related death after SARS-CoV-2 vaccination. The primary aim of this systematic review and meta-analysis was to estimate the pooled thromboembolic risk related to SARS-CoV-2 vaccines compared to placebo. The secondary outcomes included estimating the risks of arterial thromboembolism (ATE), venous thromboembolisms (VTE), hemorrhage, thrombocytopenia, and thromboembolism/hemorrhage-related death.

**Results:**

Eight RCTs of 4 vaccine platforms comprised of 195,196 participants were retrieved. SARS-CoV-2 vaccines were not associated with an increased risk of overall thromboembolism (risk ratio [RR], 1.14; 95% CI [confidence interval], 0.61–2.14; I^2^ = 35%), ATE (RR, 0.97; 95% CI, 0.46–2.06; I^2^ = 21%), VTE (RR, 1.47; 95% CI, 0.72–2.99; I^2^ = 0%), hemorrhage (RR, 0.97; 95% CI, 0.35–2.68; I^2^ = 0), and thromboembolism/hemorrhage-related death (RR, 0.53; 95% CI, 0.16–1.79; I^2^ = 0). Compared to the baseline estimated risk of these outcomes in participants administered placebos, the risk differences with vaccines were very small and not statistically significant. These findings were consistent in the subgroup analysis across 4 vaccine platforms.

**Conclusion:**

Vaccines against SARS-CoV-2 are not associated with an increased risk of thromboembolism, hemorrhage, and thromboembolism/hemorrhage-related death.

**Supplementary Information:**

The online version contains supplementary material available at 10.1186/s12959-021-00340-4.

## Introduction

Severe acute respiratory syndrome coronavirus 2 (SARS-CoV-2) has been identified as a causative agent of an emerging cluster of pneumonia in China in December 2019. Its outbreak has been declared as a pandemic leading to a global health crisis since March 11, 2020 [1]. As of August 2021, SARS-CoV-2 has infected more than 200 million individuals and caused over 4 million deaths worldwide [2]. Vaccines against SARS-CoV-2 were developed at unparalleled speeds to end the coronavirus disease 2019 (COVID-19) pandemic by controlling the viral spread.

To date, there are at least 4 vaccine platforms including mRNA, adenoviral vector, inactivated, and protein subunit vaccines that have demonstrated effectiveness in the prevention of symptomatic infection and reduction in hospitalization and mortality from COVID-19 [3–10]. Although these SARS-CoV-2 vaccines had acceptable safety profiles in phase 3 randomized controlled trials (RCTs), concerns regarding potential rare side effects including the risk of thromboembolism remain a reason for vaccine hesitancy [11]. A distinctive syndrome of vaccine-induced immune thrombotic thrombocytopenia (VITT) associated with pathogenic anti-platelet factor 4 antibodies (anti-PF4 Abs) has been reported after two adenoviral vector vaccines against SARS-CoV-2, ChAdOx1 nCoV-19 and Ad26.COV2.S [12–15]. However, this thrombotic complication linked to SARS-CoV-2 vaccines is extremely rare with an estimated incidence of 0.73 per 100,000 doses of the ChAdOx1 vaccine [16]. Most thromboembolic and hemorrhagic events after vaccination against SARS-CoV-2 are independent of anti-PF4 Abs. The risks of thromboembolism and hemorrhage after vaccination against SARS-CoV-2 remain largely unknown and have never been comprehensively evaluated in phase 3 RCTs.

Due to the rarity of thrombotic and hemorrhagic events reported in individual studies, a single RCT is underpowered to determine whether SARS-CoV-2 vaccines increase the risks of thromboembolism and hemorrhage. We conducted a systematic review and meta-analysis of phase 3 RCTs to estimate the risks of thromboembolism, hemorrhage, and death related to thrombosis or hemorrhage after vaccination against SARS-CoV-2.

## Methods

The protocol for this review was pre-specified and registered in PROSPERO (CRD42021253193). The study was subsequently conducted following Preferred Reporting Items for Systematic reviews and Meta-Analyses (PRISMA) guidelines [17]. The primary objective of this study was to estimate the risk of overall thromboembolism including arterial and venous thromboembolism of SARS-CoV-2 vaccines compared to placebo.

### Data source, search strategy and study selection

A systematic search of electronic databases was performed using PubMed, EMBASE, and Cochrane Library Database from inception to the last update on June 30, 2021 to identify RCTs reporting thromboembolic and hemorrhagic events or death related to thromboembolic and hemorrhagic events after SARS-CoV-2 vaccination. The following search terms were used: vaccine, vaccination, immunization, thromboembolism, thromboembolic, thrombosis, infarct, stroke, ischemia, ischemic, bleeding, hemorrhage, hemorrhagic, platelet, thrombocytopenia, thrombocytopenic, coagulation, coagulopathy, safety, novel coronavirus 2019, COVID-19, SARS-CoV-2, and 2019-nCoV. Additionally, studies published on the preprint server (medRxiv) and reference lists of relevant articles were manually reviewed. The inclusion criteria for eligible studies were as follows: (1) RCTs with at least 100 participants in both the vaccine and control arms, (2) reported safety outcomes which specified thromboembolic and hemorrhagic events and/or death related to thromboembolism and hemorrhage. Non-original articles (such as reviews, commentaries, or guidelines) and duplicate studies were excluded. There were no language restrictions. Two authors (N.U. and K.P.) independently searched the literature, screened titles and abstracts, and reviewed full texts to identify potentially eligible studies. Disagreements were resolved by consensus or a third reviewer (T.C.) when necessary. The selection result was reported according to the PRISMA flowchart.

### Data extraction

Two authors (N.U. and K.P.) independently reviewed data from selected studies including supplementary materials and independently extracted pre-specified data. Disagreements of extracted data were resolved by consensus or a third reviewer (T.C.) when necessary. The primary outcome was the risk of arterial and/or venous thromboembolism after vaccination against SARS-CoV-2 compared to controls. The secondary outcomes included the risks of arterial thromboembolism (ATE), venous thromboembolisms (VTE), bleeding, thrombocytopenia, and death related to thromboembolism and hemorrhage after vaccination against SARS-CoV-2.

For each study, the following data were extracted: study design, phase of clinical trials, vaccine platform (mRNA, viral vector, inactivated, or protein subunit), treatment allocation, study population, number of participants, baseline characteristics of participants (age, sex, and ethnicity), thromboembolic events, hemorrhagic events, and death related to thromboembolism or hemorrhage. Corresponding authors of the BNT162b2 study were contacted twice to request additional outcome data that were not reported. However, we were unable to obtain data from the BNT162b study.

### Quality assessment

An assessment of the methodological quality of included studies for meta-analysis was performed independently by two authors (N.U. and K.P.) using the revised version of the Cochrane risk-of-bias tool in RCTs [18]. Bias was assessed in the domains of randomization process, deviations from the intended interventions, missing outcome data, measurement of the outcomes, and selection of the reported results. Data on study characteristics and outcomes were extracted by using a standardized form. The risk of bias was graded as low, some concerns, or high. Discrepancies were resolved by consensus or contact with a third reviewer (T.C.).

### Data analysis

The meta-analysis was performed using Comprehensive Meta-Analysis software (Version 3; Biostat, Englewood, NJ, USA). The risk ratio (RR) and the risk difference of each outcome were calculated using the Mantel-Haenszel method with random-effects model and were reported as RR and risk difference (per 100,000 persons) with 95% confidence interval (95% CI) [19]. The pre-specified subgroup analyses including the risks of thromboembolism and hemorrhage across vaccine platforms (mRNA, virus vector, inactivated, or protein subunit vaccines), age groups, sex, and races would be performed if there were sufficient data. Statistical heterogeneity was assessed using I^2^ statistic which measures the inconsistency across study results. Inter-study heterogeneity was assigned as insignificant (I^2^ = 0–25%), low (I^2^ = 26–50%), moderate (I^2^ = 51–75%), or high (I^2^ > 75%) [20]. Publication bias was explored by visual inspection of the funnel plots. No formal tests for publication bias were performed as they lacked statistical power due to the low number of studies included in the meta-analysis (less than 10 studies).

## Results

The study report was prepared in accordance with the Preferred Reporting Items for Systematic Reviews and Meta-Analyses guidance (Supplementary Table S1) [17]. The PRISMA flow diagram is shown in Supplementary Fig. S1. The literature search yielded 4999 articles. After 1568 duplicates removed, a total of 3847 unique studies were screened by titles and abstracts. Of these, 3816 were excluded, and 31 full texts were screened for eligibility. Eventually 8 studies [3–10] met the eligibility criteria and were included in the qualitative and quantitative synthesis. The risk of bias in each study was individually assessed. All studies [3–10] were assigned as low risk of bias (Supplementary Fig. S2).

### Study characteristics

The main characteristics of the 8 included studies (7 published full-texts and 1 full-preprint report) are summarized in Table 1 [3–10]. The 8 studies contained 195,196 participants. A total of 104,779 participants were administered SARS-CoV-2 vaccines, and a total of 90,417 were administered placebos. There were 4 vaccine platforms including 2 mRNA vaccines (BNT162b2 and mRNA-1273) [3, 4], 3 adenoviral vector vaccines (Ad26.COV.2S, ChAdOx1 and rAD26/rAD5) [5–7], 1 inactivated vaccine (2 studies of CoronaVac) [8, 9], and 1 protein subunit vaccine (NVX-CoV23) [10]. All vaccines except the NVX-CoV23 vaccine were widely available under emergency use authorization. The majority of participants were younger than 60 years and predominantly Caucasian. Baseline characteristics including age groups, sex, races, and coexisting conditions among participants in the vaccine and the placebo groups were similar. The BNT162b2 study reported only death related to thromboembolism and hemorrhage, while the primary and other secondary outcomes were not reported. Detailed thromboembolic and hemorrhagic events of each study are summarized in Supplementary Table S2.

**Table 1:** Baseline characteristics of included randomized controlled trials

Study name	Vaccine platform	Study characteristics	Treatment allocation	Number of participants (safety data)	Age (years)	Sex (male)	Race (white, black, Asian)	Countries	Comorbidities
Polack, 2020 [3]	mRNA	Primary analysis of safety and efficacy from the phase 2/3 part of BNT162b2 in preventing symptomatic COVID-19 in persons ≥16 years Median follow-up: 2 months	BNT162b2 (30 μg), 2 doses 21 days apart	18,860 (21621)	52^a^ (Range; 16–89)	51.1%	82.9, 9.2, 4.2%	US 76.7%, Argentina 15.3%, Brazil 6.1%	Diabetes 8.3%, chronic lung disease 7.8%, cancers 3.9%
			Saline	18,846 (21631)	52^a^ (Range; 16–91)	50.1%	82.9, 9.4, 4.3%	US 76.7%, Argentina 15.3%, Brazil 6.0%	Diabetes 8.4%, chronic lung disease 7.7%, cancers 3/5%
Baden, 2020 [4]	mRNA	Primary analysis of safety and efficacy of phase 3 RCT in preventing COVID-19 in persons ≥18 years Median follow-up: 63 days	mRNA-1273 (100 μg), 2 doses 28 days apart	15,170 (15166)	51.3^b^ (Range; 18–95)	52.2%	79.2, 10.3, 4.3%	US 100%	Diabetes 9.5%, severe obesity 6.8%, cardiac disease 5%, chronic lung disease 4.7%
			Saline	15,181 (15185)	51.4^b^ (Range; 18–95)	53.1%	79.1, 10.1, 4.8%	US 100%	Diabetes 9.5%, severe obesity 6.7%, cardiac disease 4.9%, chronic lung disease 4.9%
Sadoff, 2021 [5]	Adenoviral vector	Primary analysis of safety and efficacy of phase 3 RCT in preventing COVID-19 in persons ≥18 years Median follow-up: 58 days	Ad26.COV2.S (A single dose of 5 × 10^10^ viral particles)	21,895	52^a^ (Range; 19–100)	55.1%	58.7, 19.4, 3.4%	US 44.1%, Latin America 40.9%, South Africa 15.0%	Obesity 28.7%, hypertension 10.2%, diabetes 7.8%
			Saline	21,888	52^a^ (Range; 18–94)	54.7%	58.7, 19.5, 3.1%	US 44.1%, Latin America 40.9%, South Africa 15.0%	Obesity 28.4%, hypertension 10.5%, diabetes 7.7%
Voysey, 2021 [6]	Adenoviral vector	Interim analysis of 4 cohorts of phase 1/2/3 RCT parts in preventing COVID-19 in persons ≥18 years Median follow-up: 3.4 months	ChAdOx1 nCoV-19 (2.2–6.5 × 10^10^ viral particles, 2 doses 4- ≥12 weeks apart)	12,021	Age (18–55 years) 81.5%	44.2%	75.1, 10.0, 3.7%	UK 50.0%, Brazil 41.6%, South Africa 8.4%	Cardiovascular disease 12.6%, respiratory disease 9.9%, diabetes 2.8%
			Meningococcal group A, C, W, and Y conjugate vaccine or saline	11,724	Age (18–55 years) 83.5%	44.1%	75.4, 10.2, 3.3%	UK 48.8%, Brazil 42.7%, South Africa 8.5%	Cardiovascular disease 12.0%, respiratory disease 10.0%, diabetes 2.5%
Logunov, 2021 [7]	Adenoviral vector	Preliminary efficacy and safety analysis of phase 3 RCT in preventing COVID-19 in persons ≥18 years Median follow-up: 48 days	rAd26 (1st dose) and rAd5 (2nd dose) containing 1 × 10^11^ viral particles, 2 doses 21 days apart	14,964 (16427)	45.3^b^ (SD 12.0)	61.1%	98.5%, NA, 1.5%	Russia 100%	Diabetes, hypertension, ischemic heart disease, obesity 24.7%
			Excipients	4902 (5435)	45.3^b^ (SD 11.9)	61.5%	98.5%, NA, 1.5%	Russia 100%	Diabetes, hypertension, ischemic heart disease, obesity 25.2%
Tanriover, 2021 [8]	Inactivated	Interim analysis of efficacy and safety of phase 3 RCT in preventing COVID-19 in persons aged 18–59 years Median follow-up: 43 days	CoronaVac (3 μg of SARS-CoV-2 virions), 2 doses 14 days apart	6646 (6648)	45^a^ (IQR; 35–51)	57.4%	NA	Turkey 100%	Hypertension 11.8%, diabetes 4.9%, chronic lung disease 2.9%
			Aluminium hydroxide diluent	3568 (3568)	45^a^ (IQR;37–51)	58.65	NA	Turkey 100%	Hypertension, 11.6%, diabetes 4.5%, chronic lung disease 2.9%
Palacios, 2021 [9] (preprint)	Inactivated	Interim analysis of efficacy and safety of phase 3 RCT in preventing COVID-19 in healthcare workers ≥18 years Median follow-up: 2 months after the second dose	CoronaVac (3 μg of SARS-CoV-2 virions), 2 doses 14 days apart	6195 (6202)	39.4^b^ (SD 10.7)	36.6%	75.8, 5.3, 2.4%	Brazil 100%	Obesity 22.4%, cardiovascular disease 12.8%, diabetes 3.5%
			Aluminium hydroxide diluent	6201 (6194)	39.6^b^ (SD 10.8)	35.0%	74.8, 5.2, 2.6%	Brazil 100%	Obesity 22.6%, cardiovascular disease 12.5%, diabetes 3.2%
Heath, 2021 [10]	Protein subunit	Interim analysis of efficacy and safety of phase 3 RCT in preventing COVID-19 in persons ≥18 years Median follow-up: 66 days after the first dose and 45 days after the second dose	NVX-CoV2373 (5 μg), 2 doses 21 days apart	7020 (7569)	56^a^ (18–84)	51.4%	94.4, 0.4, 2.9%	UK 100%	Chronic lung, cardiac, renal, neurologic, hepatic, immunocompromising conditions, and obesity 44.4%
			Saline	7019 (7570)	56^a^ (18–64)	51.7%	94.5, 0.4, 3.0%	UK 100%	Chronic lung, cardiac, renal, neurologic, hepatic, immunocompromising conditions, and obesity 44.8%

^a^Median; ^b^Mean; COVID-19, coronavirus disease 2019; *RCT* randomized controlled trial; *US* United States; *UK* United Kingdom

### The risk of thromboembolism after vaccination against SARS-CoV-2

**Fig. 1:**
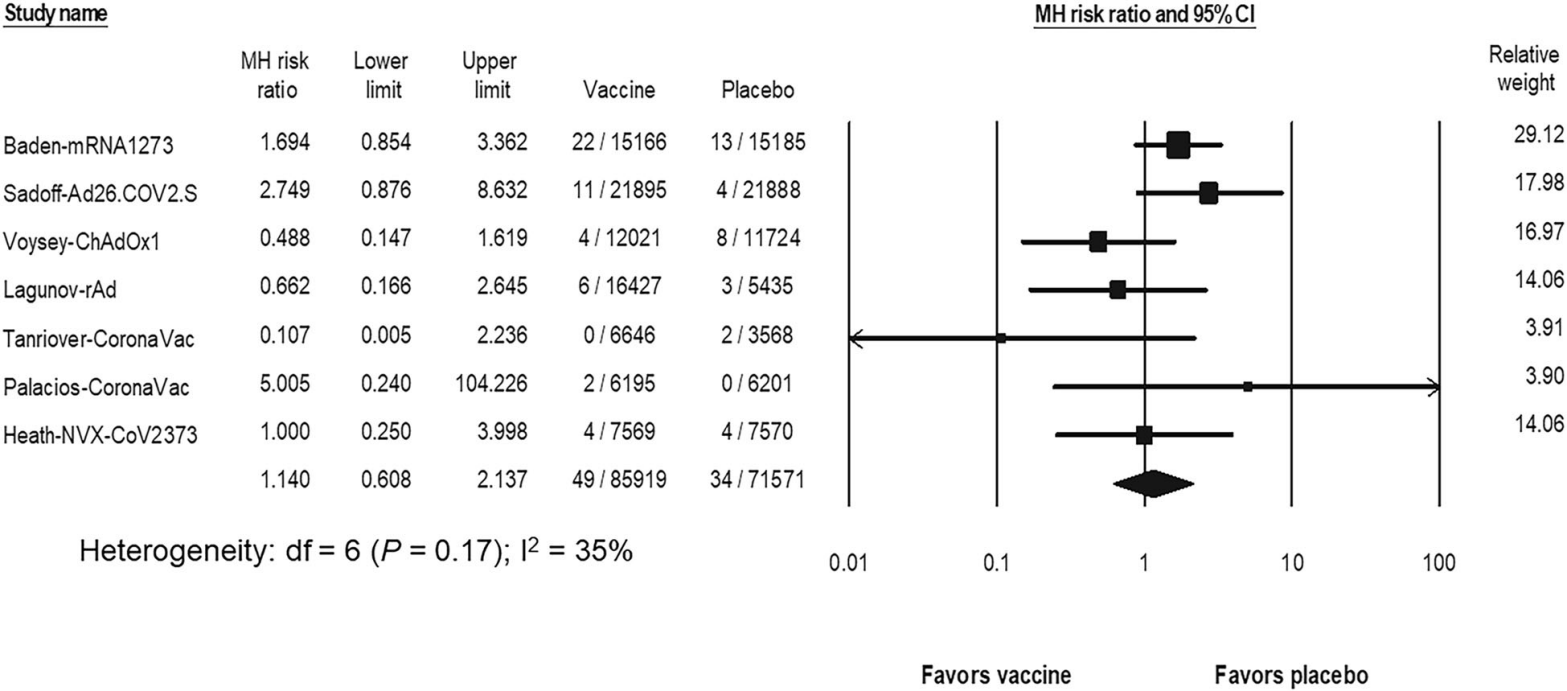
The pooled risk ratio of overall thromboembolism between the SARS-CoV-2 vaccine and the placebo groups

The estimated risk of thromboembolism including arterial and/or venous thrombosis was estimated from 7 of the 8 studies, while omitting the BNT162b study [4–10]. A total of 85,915 and 71,571 participants received either a vaccine or placebo, respectively. The pooled RR of thromboembolism after vaccination was 1.14 (95% CI, 0.61 to 2.14; I^2^ = 35%) (Fig. 1). With a baseline estimated risk of thromboembolism in the placebo group of 52 events per 100,000 persons (95% CI, 33 to 83; I^2^ = 37%), the risk difference with the vaccine group was 7.8 events per 100,000 persons (95% CI, − 20 to 36; I^2^ = 33%). The subgroup analysis did not demonstrate an increased risk of thromboembolism in any vaccine platform (*P* = 0.80) (Supplementary Fig. S3).

### The risks of arterial thromboembolism and venous thromboembolism after vaccination against SARS-CoV-2

**Fig. 2:**
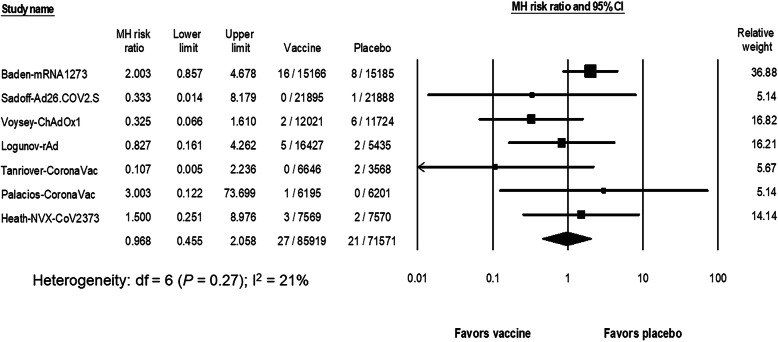
The pooled risk ratio of arterial thromboembolism between the SARS-CoV-2 vaccine and the placebo groups

The risks of ATE and VTE after SARS-CoV-2 vaccination were estimated from the same 7 studies, again excluding the BNT162b2 study [4–10]. No VTE events occurred in one inactivated vaccine study (Tanriover) [8]. The pooled RR of ATE after SARS-CoV-2 vaccination was 0.97 (95% CI, 0.46 to 2.06; I^2^ = 21%) (Fig. 2). With an estimated risk of ATE from 7 studies [4–10] in the placebo group of 38 events per 100,000 persons (95% CI, 23 to 63; I^2^ = 20%), the risk difference with the vaccine group was − 1.8 events per 100,000 persons (95% CI, − 20 to 17; I^2^ = 27%).

**Fig. 3:**
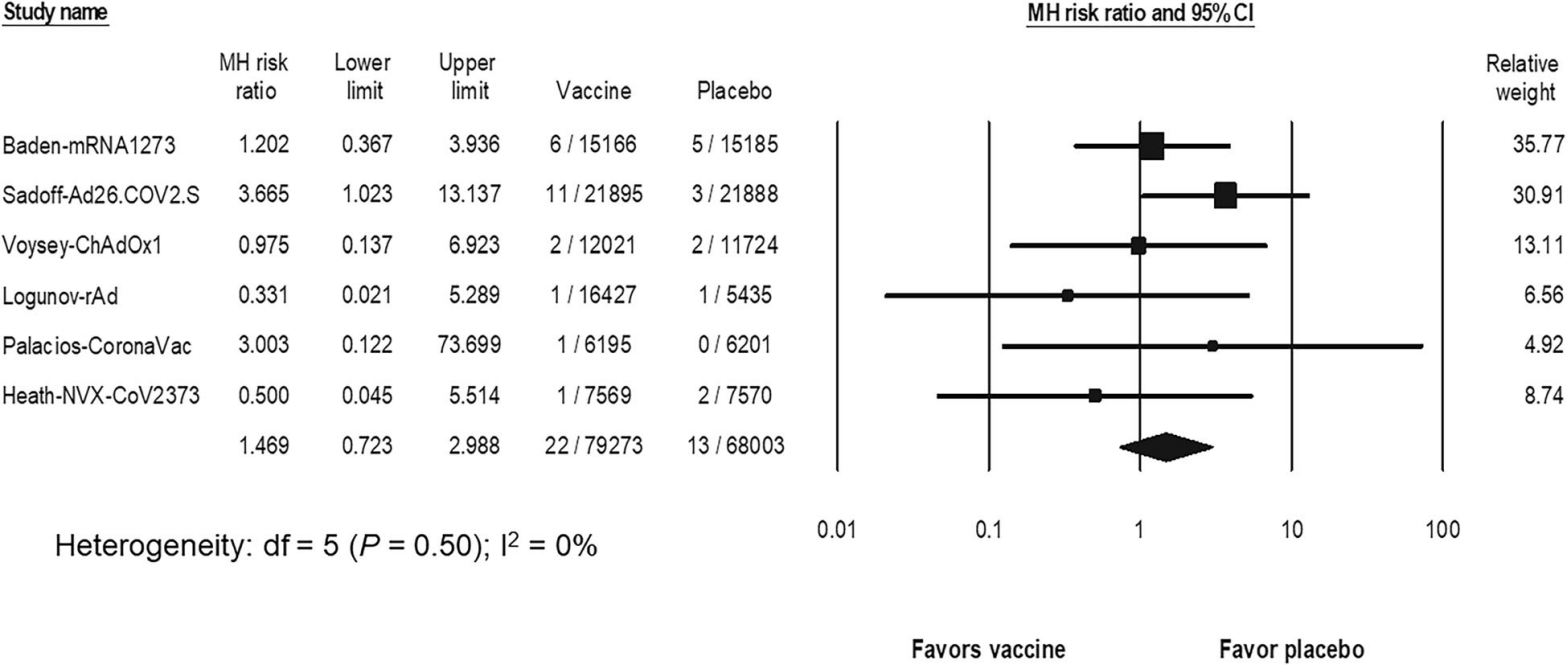
The pooled risk ratio of venous thromboembolism between the SARS-CoV-2 vaccine and the placebo groups

The RR of VTE after SARS-CoV-2 vaccination (6 studies [4–7, 9, 10]; *N* = 79,273 in the vaccine group and *N* = 68,003 in the placebo group) was 1.47 (95% CI, 0.72 to 2.99; I^2^ = 0%) (Fig. 3). The risk of VTE after SARS-CoV-2 vaccination was increased only in the Ad26.COV2.S study (RR = 3.67; 95% CI, 1.02 to 13.14). With an estimated risk of VTE from 7 studies [4–10] in the placebo group of 21 events per 100,000 persons (95% CI, 13 to 36; I^2^ = 0), the risk difference with the vaccine group was 6.3 events per 100,000 persons (95% CI, − 8.5 to 21; I^2^ = 0). The subgroup analysis did not demonstrate an increased risk of either ATE or VTE in any vaccine platform, including the adenoviral vector subtype (*P* = 0.23 and *P* = 0.81, respectively) (Supplementary Fig. S4 and Fig. S5).

### The risks of hemorrhage and thrombocytopenia after vaccination against SARS-CoV-2

**Fig. 4:**
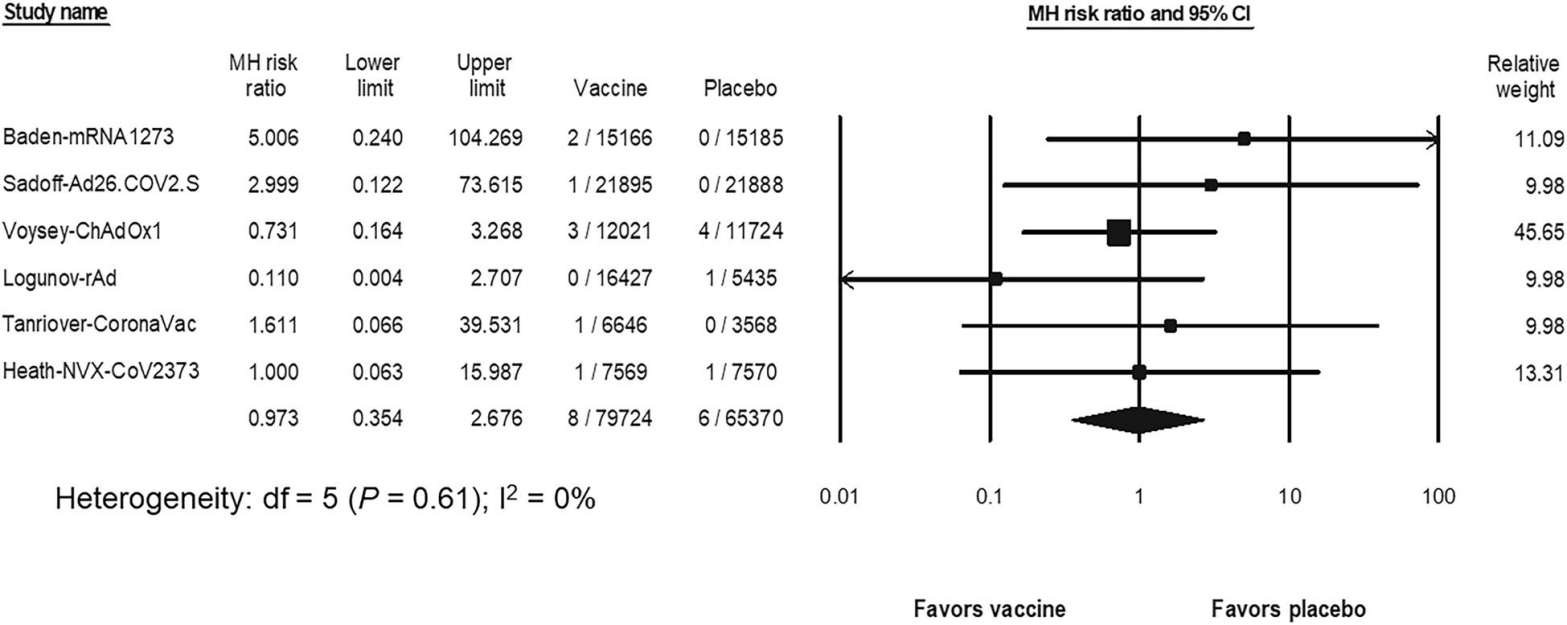
The pooled risk ratio of hemorrhage between the SARS-CoV-2 vaccine and the placebo groups

The risk of hemorrhage was estimated from 7 studies while excluding the BNT162b2 study (*N* = 85,919 in the vaccine group and *N* = 71,751 in the placebo group) [4–10]. No bleeding events occurred in one inactivated vaccine study (Palacios) [9]. The RR of hemorrhage after SARS-CoV-2 vaccination (6 studies [4–8, 10] included 79,724 participants in the vaccine group and 65,370 participants in the placebo group) was 0.97 (95% CI, 0.35 to 2.68, I^2^ = 0%) (Fig. 4). With an estimated risk of bleeding from 7 studies [4–10] in the placebo group of 18 events per 100,000 persons (95% CI, 8 to 35; I^2^ = 0), the risk difference with the vaccine group was 4.1 events per 100,000 persons (95% CI, − 5.3 to 13.5; I^2^ = 0). The subgroup analysis did not demonstrate an increased risk of bleeding in any vaccine platform (*P* = 0.68) (Supplementary Fig. S6).

The risk of thrombocytopenia after SARS-CoV-2 vaccination was not analyzed because no events were reported in the included studies.

### The risk of death related to thromboembolic and hemorrhagic events after vaccination against SARS-CoV-2

**Fig. 5:**
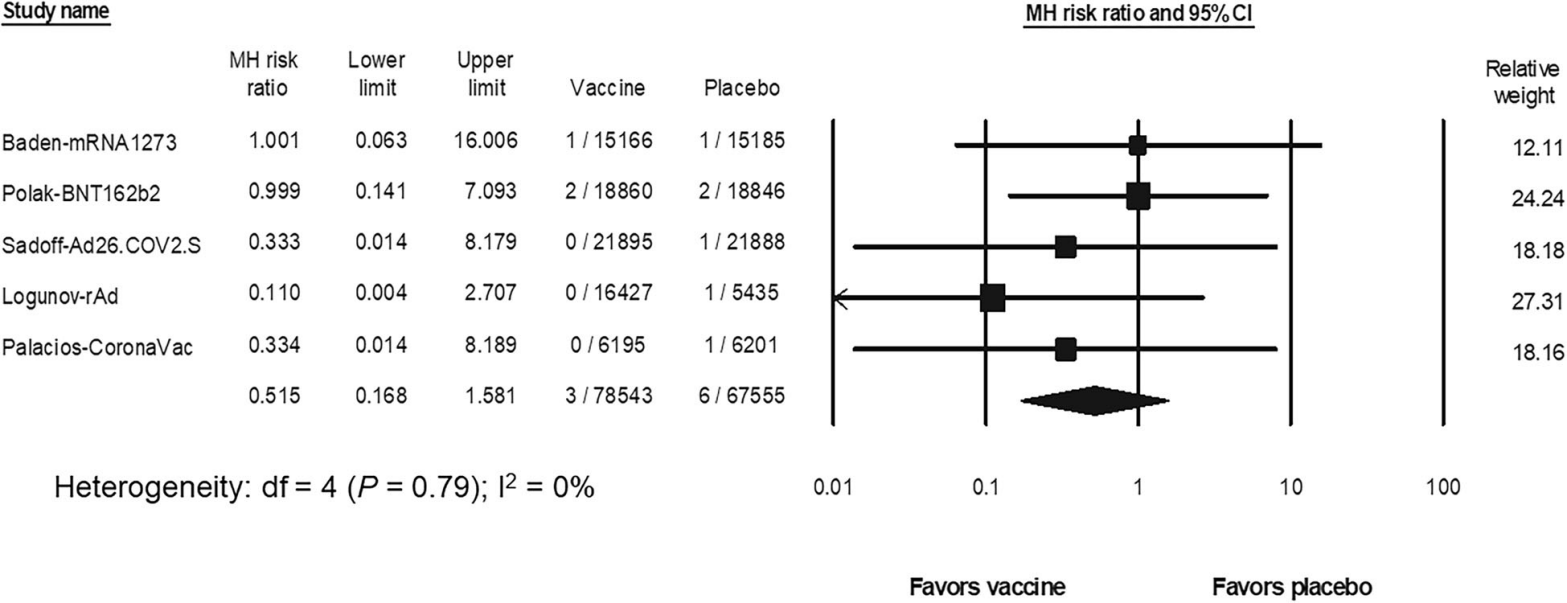
The pooled risk ratio of death related to thromboembolism and hemorrhage between the SARS-CoV-2 vaccine and the placebo groups

The risk of death related to thromboembolism and hemorrhage was estimated from all 8 studies [3–10] (*N* = 104,779 in the vaccine group and *N* = 90,417 in the placebo group). No deaths related to thromboembolism or bleeding occurred in the ChAdOx1 study, one CoronaVac study (Tanriover) and the NVX-CoV23 study 6, 8, 10]. The RR of death from thromboembolism or hemorrhage after SARS-CoV-2 vaccination (5 studies [3–5, 7, 9] included 78,543 participants in the vaccine group and 67,555 participants in the placebo group) was 0.53 (95% CI, 0.16 to 1.79; I^2^ = 0%) (Fig. 5). With an estimated risk of thromboembolism/hemorrhage-related death from 8 studies [3–10] in the placebo group of 9 events per 100,000 persons (95% CI, 5 to 19; I^2^ = 0), the risk difference with the vaccine group was − 3.7 events per 100,000 persons (95% CI, − 12.2 to 4.8; I^2^ = 0). The subgroup analysis did not demonstrate an increased risk of death related to thromboembolism and hemorrhage in any vaccine platform (*P* = 0.48) (Supplementary Fig. S7).

## Discussion

In this systematic review and meta-analysis of 8 RCTs involving nearly 200,000 participants, the risks of overall thromboembolism, ATE, VTE, hemorrhage, and death related to thromboembolism and hemorrhage were not significantly increased with vaccination against SARS-CoV-2. An increased risk of VTE was observed only with the Ad26.COV2.S vaccine. The absolute risk differences of all outcomes were less than 0.01% or less than 10 per 100,000 persons. This meta-analysis confirmed the rarity of thromboembolic and hemorrhagic events after SARS-CoV-2 vaccines observed in phase 3 RCTs across all vaccine platforms.

In our systematic review, there were no thrombocytopenia events reported in the included studies suggesting the rarity of significant thrombocytopenia after SARS-CoV-2 vaccination. Of note, mild asymptomatic thrombocytopenia may be undetected and underreported in clinical trials. According to population-based studies, the risk of unspecified thrombocytopenia after ChAdOx1 vaccination was increased in Denmark and Norway [21] and in England [22], while the risk of immune thrombocytopenia after ChAdOx1 vaccination was increased in Scotland and Thailand [23, 24]. Due to very low prevalence, this meta-analysis remained underpowered to detect the risk of thrombocytopenia after SARS-CoV-2 vaccination.

The subgroup analysis to determine the risks of thromboembolism and hemorrhage across vaccine platforms was performed. Compared to placebo, each vaccine platform did not show an increase in the risks of thromboembolism and hemorrhage in all analyses. These findings support the general safety among different SARS-CoV-2 vaccine platforms. There were insufficient data to perform other pre-specified subgroup analysis including age, gender and race.

COVID-19 is associated with the risks of systemic coagulopathy, thrombosis, and bleeding, especially in critically ill patients [22, 25–29]. Since SARS-CoV-2 vaccines can effectively prevent symptomatic infection, hospitalization, and mortality [3–10], the risks of thromboembolism, hemorrhage, and death related to thromboembolism and hemorrhage from COVID-19 may also be reduced in vaccinated participants. However, in most studies, the SARS-CoV-2 infection status was not specified in participants who experienced thromboembolic or hemorrhagic events. A contributing risk of COVID-19 associated thromboembolism and hemorrhage could not be totally excluded.

The risks of thromboembolism and hemorrhage were also assessed in large national cohort studies. Although there was no association between the ChAdOx1 vaccine and thromboembolism in the phase 3 RCT, some national cohorts have observed an increased risk of thromboembolism after ChAdOx1 vaccination. The population-based studies from Denmark and Norway, which included 281,264 people vaccinated with the ChAdOx1 vaccine, demonstrated an increased rate of VTE and cerebral vein thrombosis, but not for ATE, and a small increased risk of unspecified thrombocytopenia among recipients of the ChAdOx1 vaccine [21]. The larger cohort from England, which included 19,608,008 people vaccinated with the ChAdOx1 vaccine, also demonstrated an increased risk of unspecified thrombocytopenia, VTE, cerebral vein thrombosis, and rare arterial thromboembolic events, but not for overall ATE, stroke, and myocardial infarction, within 28 days after ChAdOx1 vaccination [22]. However, both cohorts did not specify the numbers of VITT cases, which might have been included in both cohorts [12–16]. It remains uncertain whether the risks of VTE and thrombocytopenia after ChAdOx1 vaccination would be different from these reports if VITT cases were excluded. In contrast, the Scottish population-based study, which included 1.71 million people vaccinated with the ChAdOx1 vaccine, demonstrated an increased risk of immune thrombocytopenia and a minimally increased risk of ATE among recipients of the ChAdOx1 vaccine. Notably, there was no association between ChAdOx1 vaccination and VTE including cerebral vein thrombosis [23]. The reasons underlying different thromboembolic risks observed among these national cohorts remained unclear. Since the young age group is at higher risk for VITT, while the elderly age group is at higher risk for thromboembolism, asymmetrical age distribution of vaccine recipients among cohorts may be an important confounder. In the Scandinavian cohort, the ChAdOx1 vaccine was administered to people aged between 18 to 65 years [21]. Similarly, the ChAdOx1 vaccine was vaccinated in a higher proportion of people aged younger than 60 years in England during the study period [22]. In contrast, the highest uptake for the ChAdOx1 vaccine was found in people aged older than 65 years in Scotland [30]. Additionally, there is the potential of an overestimation of vaccine-associated adverse events with the low incidence in most study designs to assess vaccine safety [31].

Although a minimally increased risk of VTE was observed in the Ad26.COV2.S vaccine arm in the phase 3 RCT, there were very few thromboembolic events observed among 288,368 recipients of the Ad26.COV2.S vaccine in South Africa [32]. No VITT or thrombocytopenia were documented in the South African cohort.

Thromboembolic events were not specified in the BNT162b study. The Scottish population-based study, which included 0.82 million people vaccinated with the BNT162b vaccine, demonstrated no increased risks of thrombocytopenia, VTE and ATE among recipients of the BNT162b vaccine [23]. Similarly, the population-based study from Israel demonstrated no increased risks of thromboembolism, hemorrhage, and thrombocytopenia after BNT162b2 vaccination [25]. In contrast, the cohort from England, which included 9,513,625 people vaccinated with the BNT162b vaccine, demonstrated an increased risk of ATE, ischemic stroke and cerebral vein thrombosis, but not for thrombocytopenia and VTE [22]. Whether the risks of thromboembolism and thrombocytopenia might be increased after BNT162b vaccination remains undetermined due to the conflicting findings among national cohort studies. One should also be cautious to generalize the risks of thromboembolism, hemorrhage, and thrombocytopenia after SARS-CoV-2 vaccination due to different genetic and thromboembolic risks among populations.

The strength of a meta-analysis of large multinational phase 3 RCTs is its inherent advantage to have even distribution of demographic characteristics, comorbidities, especially thrombotic and bleeding risk factors, and other unmeasured covariates. All RCTs included in this meta-analysis were assessed to have low risk of bias. The included studies had insignificant or low statistical heterogeneity suggesting that the effects can reasonably be combined in a meta-analysis.

There are some limitations of this study. Although it aggregated approximately 100,000 vaccinated participants, it may be insufficient to document extremely rare events such as significant thrombocytopenia. Additionally, despite our attempts to contact the authors, we were not able to obtain the data from the BNT162b2 study, and therefore this study was not included in most analyses. Consequently, there was only one type of vaccine in the mRNA, inactivated, and protein subunit platform in subgroup analysis. The lack of detailed descriptions of participants who experienced thromboembolic or hemorrhagic events also precluded evaluation of several other important subgroups analysis such as age, sex, race, coexisting comorbidities, and COVID-19 status. Asymmetrical age distribution of vaccine recipients and potentially incomplete adjustment for covariates, especially cardiovascular comorbidities and genetic factors, may result in different estimated thromboembolic risks among population-based studies. It may require the pooled analysis of large national cohorts to have sufficient power to determine the risks of thromboembolism and hemorrhages after vaccination against SARS-CoV-2 and risks among different vaccine platforms. Incomplete data entry and a lack of adequate adjustment for baseline confounders would potentially make such analysis very challenging.

## Conclusions

This meta-analysis demonstrated no increased risks of thromboembolism, hemorrhage, and death from thromboembolism and hemorrhage after vaccination against SARS-CoV-2 across all vaccine platforms. These findings provide information that may assist global vaccination campaigns in order to reduce vaccine hesitancy. Although marginal risks of thromboembolism and hemorrhage in some subgroup populations may not be excluded, the estimated incidence of these events was very low. The absolute risks of thromboembolism and hemorrhage of SARS-CoV-2 vaccines were very small in the context of the proven benefits of vaccination against SARS-CoV-2 and the globally high incidence of severe and fatal cases of SARS-CoV-2 infection.

## Supplementary Information

**Additional file 1.**

## Data Availability

Not applicable.

## Abbreviations

ATE: arterial thromboembolism
CI: confidence interval
COVID-19: coronavirus disease 2019
PRISMA: Preferred Reporting Items for Systematic reviews and Meta-Analyses
RCT: randomized controlled trial
RR: risk ratio
SARS-CoV-2: severe acute respiratory syndrome coronavirus 2
VITT: vaccine-induced immune thrombotic thrombocytopenia
VTE: venous thromboembolism
