# Supplementary material for: Thromboembolic and hemorrhagic risks after vaccination against SARS-CoV-2: a systematic review and meta-analysis of randomized controlled trials
Source: Thromb J. 2021 Nov 13;19:86. doi: 10.1186/s12959-021-00340-4 (PMC8590131; doi:10.1186/s12959-021-00340-4)
Supplement: Supplementary file 1 — Additional file 1. [file 12959_2021_340_MOESM1_ESM.pdf]

Supplemental materials

Table S1 PRISMA checklist

Section/topic	#	Checklist item	Reported on page #
**TITLE**			
Title	1	Identify the report as a systematic review, meta-analysis, or both.	1
**ABSTRACT**			
Structured summary	2	Provide a structured summary including, as applicable: background; objectives; data sources; study eligibility criteria, participants, and interventions; study appraisal and synthesis methods; results; limitations; conclusions and implications of key findings; systematic review registration number.	2-3
**INTRODUCTION**			
Rationale	3	Describe the rationale for the review in the context of what is already known.	4-5
Objectives	4	Provide an explicit statement of questions being addressed with reference to participants, interventions, comparisons, outcomes, and study design (PICOS).	5
**METHODS**			
Protocol and registration	5	Indicate if a review protocol exists, if and where it can be accessed (e.g., Web address), and, if available, provide registration information including registration number.	5
Eligibility criteria	6	Specify study characteristics (e.g., PICOS, length of follow-up) and report characteristics (e.g., years considered, language, publication status) used as criteria for eligibility, giving rationale.	5-6
Information sources	7	Describe all information sources (e.g., databases with dates of coverage, contact with study authors to identify additional studies) in the search and date last searched.	5-6
Search	8	Present full electronic search strategy for at least one database, including any limits used, such that it could be repeated.	5-6
Study selection	9	State the process for selecting studies (i.e., screening, eligibility, included in systematic review, and, if applicable, included in the meta-analysis).	5-6
Data collection process	10	Describe method of data extraction from reports (e.g., piloted forms, independently, in duplicate) and any processes for obtaining and confirming data from investigators.	6-7

Data items	11	List and define all variables for which data were sought (e.g., PICOS, funding sources) and any assumptions and simplifications made.	6-7
Risk of bias in individual studies	12	Describe methods used for assessing risk of bias of individual studies (including specification of whether this was done at the study or outcome level), and how this information is to be used in any data synthesis.	7
Summary measures	13	State the principal summary measures (e.g., risk ratio, difference in means).	7-8
Synthesis of results	14	Describe the methods of handling data and combining results of studies, if done, including measures of consistency (e.g., I^2) for each meta-analysis.	7-8

Section/topic	#	Checklist item	Reported on page #
Risk of bias across studies	15	Specify any assessment of risk of bias that may affect the cumulative evidence (e.g., publication bias, selective reporting within studies).	7-8
Additional analyses	16	Describe methods of additional analyses (e.g., sensitivity or subgroup analyses, meta-regression), if done, indicating which were pre-specified.	7-8
**RESULTS**			
Study selection	17	Give numbers of studies screened, assessed for eligibility, and included in the review, with reasons for exclusions at each stage, ideally with a flow diagram.	8 and supp figure S1
Study characteristics	18	For each study, present characteristics for which data were extracted (e.g., study size, PICOS, follow-up period) and provide the citations.	8-9
Risk of bias within studies	19	Present data on risk of bias of each study and, if available, any outcome level assessment (see item 12).	8
Results of individual studies	20	For all outcomes considered (benefits or harms), present, for each study: (a) simple summary data for each intervention group (b) effect estimates and confidence intervals, ideally with a forest plot.	9-11
Synthesis of results	21	Present results of each meta-analysis done, including confidence intervals and measures of consistency.	9-11
Risk of bias across studies	22	Present results of any assessment of risk of bias across studies (see Item 15).	9-11
Additional analysis	23	Give results of additional analyses, if done (e.g., sensitivity or subgroup analyses, meta-regression [see Item 16]).	9-11
**DISCUSSION**			
Summary of evidence	24	Summarize the main findings including the strength of evidence for each main outcome; consider their relevance to key groups (e.g., healthcare providers, users, and policy makers).	9-15

Limitations	25	Discuss limitations at study and outcome level (e.g., risk of bias), and at review-level (e.g., incomplete retrieval of identified research, reporting bias).	15-16
Conclusions	26	Provide a general interpretation of the results in the context of other evidence, and implications for future research.	16
**FUNDING**			
Funding	27	Describe sources of funding for the systematic review and other support (e.g., supply of data); role of funders for the systematic review.	NA

From: Moher D, Liberati A, Tetzlaff J, Altman DG, The PRISMA Group (2009). Preferred Reporting Items for Systematic Reviews and Meta-Analyses: The PRISMA Statement. PLoS Med 6(6): e1000097. doi:10.1371/journal.pmed1000097

NA, not applicable; Supp, supplementary

Table S2 Thromboembolic and hemorrhagic events and death related to thromboembolic and hemorrhagic events of individual randomized controlled trials

Study name	Vaccine platform	Treatment allocation	Number of participants (safety data)	Thromboembolism	ATE	VTE	Hemorrhage	Death related to thromboembolism and hemorrhage
Polack,[3] 2020	mRNA	BNT162b2	18860 (21621)	NA	NA	NA	NA	2 (atherosclerosis; cardiac arrest)
		Saline	18846 (21631)	NA	NA	NA	NA	2 (hemorrhagic stroke, AMI)
Baden,[4] 2020	mRNA	mRNA-1273	15170 (15166)	22 (11 CAD; 5 CVA; 4 PE; 2 DVT)	16 (11 CAD; 5 CVA)	6 (4 PE; 2 DVT)	2 (2 subdural hematoma)	1 (cardiopulmonary arrest)
		Saline	15181 (15185)	13 (7 CAD; 1 CVA; 5 PE)	8 (7 CAD; 1 CVA)	5 (5 PE)	0	1 (cardiopulmonary arrest)
Sadoff,[5] 2021	Adenoviral vector	Ad26.COV2.S	21895	11 (6 DVT; 4 PE; 1 CVT)	0	11 (6 DVT; 4 PE; 1 CVT)	1 (cerebral hemorrhage)	0
		Saline	21888	4 (1 CAD; 2 DVT; 1 PE)	1 (1 CAD)	3 (2 DVT; 1 PE)	0	1 (CAD)
Voysey,[6] 2021	Adenoviral vector	ChAdOx1 nCoV-19	12021	4 (1 CAD; 1 CVA; 1 PE; 1 unspecified thrombosis)	2 (1 CAD; 1 CVA)	2 (1 PE; 1 unspecified)	3 (1 GI; 2 GU)	0
		Meningococcal group A, C, W, and Y conjugate vaccine or saline	11724	8 (1 CAD; 5 CVA; 1 DVT; 1 thrombophlebitis)	6 (1 CAD; 5 CVA)	2 (1 DVT; 1 thrombophlebitis)	4 (1 subarachnoid hemorrhage; 1 GI; 1 GU; 1 hemoptysis)	0
Logunov,[7] 2021	Adenoviral vector	rAd26 (1st dose) and rAd5 (2nd dose)	14964 (16427)	6 (2 CAD; 3 CVA; 1 DVT)	5 (2 CAD; 3 CVA)	1 (1 DVT)	0	0
		Excipients	4902 (5435)	3 (1 CAD; 1 CVA; 1)	2 (1 CAD; 1 CVA)	1 (1 thrombophlebitis)	1 (1 hemorrhagic stroke)	1 (1 hemorrhagic stroke)

Tanriover,[8] 2021	Inactivated	CoronaVac	6646 (6648)	0	0	0	1 (1 GI)	0
		Aluminium hydroxide diluent	3568 (3568)	2 (1 CAD; 1 CVA)	2 (1 CAD; 1 CVA)	0	0	0
Palacios,[9] 2021 (preprint)	Inactivated	CoronaVac	6195 (6202)	2 (1 CVA; 1 DVT)	1 (1 CVA)	1 (1 DVT)	0	0
		Aluminiun hydroxide diluent	6201 (6194)	0	0	0	0	1 (cardiopulmonary arrest)
Heath,[10] 2021	Protein subunit	NVX-CoV2373	7020 (7569)	4 (3 CAD; 1 PE)	3 (3 CAD)	1 (1 PE)	1 (1 GI)	0
		Saline	7019 (7570)	4 (1 CVA; 1 PAD; 2 PE)	2 (1 CVA; 1 PAD)	2 (2 PE)	1 (1 epistaxis)	0

CAD, coronary artery disease (including acute coronary syndrome, acute myocardial infarction and myocardial ischemia); CVA, cerebrovascular accident (including stroke and transient ischemic attack); CVT, cerebral vein thrombosis; DVT, deep vein thrombosis; GI, gastrointestinal; GU, genitourinary; PAD, peripheral arterial disease (including peripheral ischemia); PE, pulmonary embolism

Table S3 The estimated baseline risks of each outcome in the vaccine and in the placebo groups using random-effects model

Outcome	The estimated baseline risk in the vaccine group (per 100,000 persons [95% confidence interval])	The estimated baseline risk in the placebo group (per 100,000 persons [95% confidence interval])
Overall thromboembolism	50 (27-92)	52 (33-83)
Arterial thromboembolism	27 (11-65)	38 (23-63)
Venous thromboembolism	27 (15-48)	21 (13-36)
Hemorrhage	14 (7-27)	18 (8-35)
Death related to thromboembolisms or hemorrhage	9 (4-18)	9 (5-19)

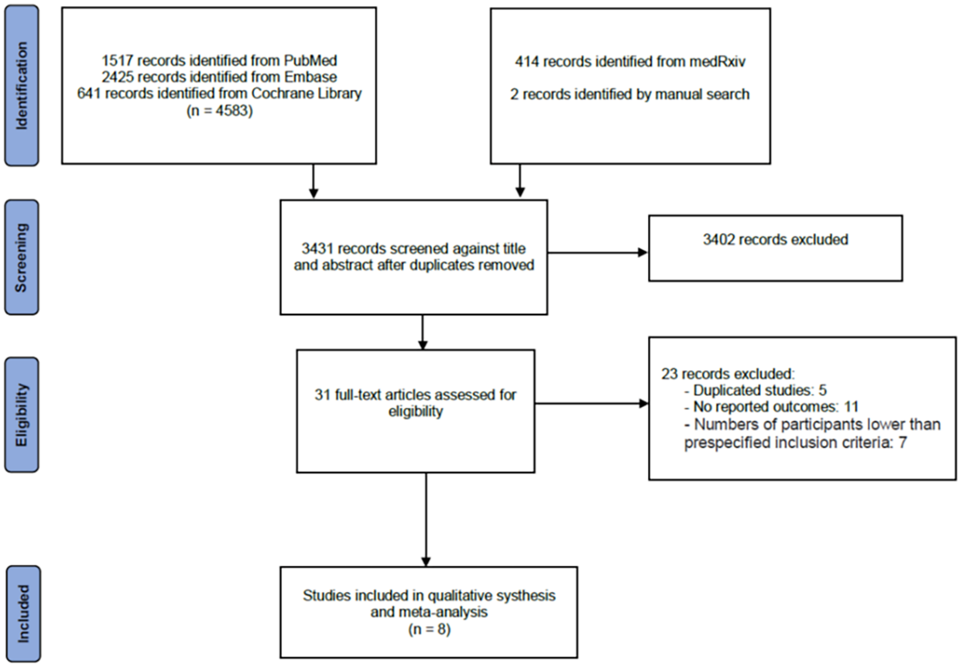

Figure S1 The PRISMA flow diagram

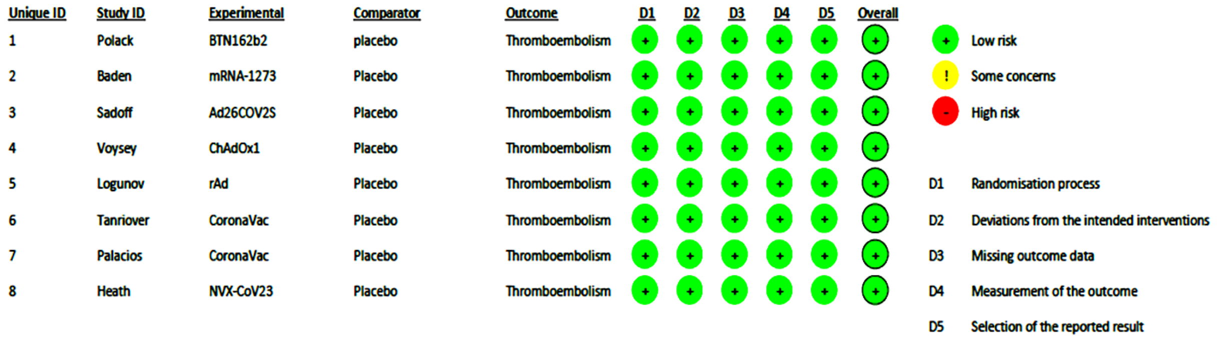

Figure S2 The risk of bias assessment

Study name	Subgroup within study	MH risk ratio	Lower limit	Upper limit	Vaccine	Placebo	Relative weight
Tanriover-CoronaVac	Inactivated	0.107	0.005	2.236	0 / 6646	2 / 3568	50.00
Palacios-CoronaVac	Inactivated	5.005	0.240	104.226	2 / 6195	0 / 6201	50.00
		0.733	0.017	31.658	2 / 12841	2 / 9769	
Baden-mRNA1273	mRNA	1.694	0.854	3.362	22 / 15166	13 / 15185	100.00
		1.694	0.854	3.362	22 / 15166	13 / 15185	
Heath-NVX-CoV2373	Protein subunit	1.000	0.250	3.998	4 / 7569	4 / 7570	100.00
		1.000	0.250	3.998	4 / 7569	4 / 7570	
Sadoff-Ad26.COV2.S	Viral vector	2.749	0.876	8.632	11 / 21895	4 / 21888	35.58
Voysey-ChAdOx1	Viral vector	0.488	0.147	1.619	4 / 12021	8 / 11724	34.26
Lagunov-rAd	Viral vector	0.662	0.166	2.645	6 / 16427	3 / 5435	30.16
		0.989	0.329	2.972	21 / 50343	15 / 39047	
		1.361	0.800	2.314	49 / 85919	34 / 71571	

Heterogeneity: df = 3 (P = 0.80)

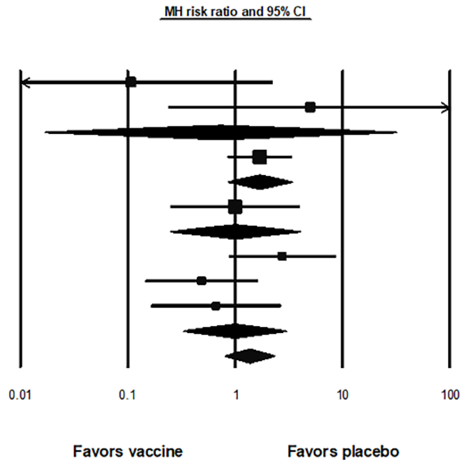

Figure S3 The subgroup analysis to estimate the risk ratio of overall thromboembolism between the SARS-CoV-2 vaccine and the placebo groups and across vaccine platforms

Study name	Subgroup within study	MH risk ratio	Lower limit	Upper limit	Vaccine	Placebo	Relative weight
Tanriover-CoronaVac	Inactivated	0.107	0.005	2.236	0 / 6646	2 / 3568	51.20
Palacios-CoronaVac	Inactivated	3.003	0.122	73.699	1 / 6195	0 / 6201	48.80
		0.546	0.021	14.284	1 / 12841	2 / 9769	
Baden-mRNA1273	mRNA	2.003	0.857	4.678	16 / 15166	8 / 15185	100.00
		2.003	0.857	4.678	16 / 15166	8 / 15185	
Heath-NVX-CoV2373	Protein subunit	1.500	0.251	8.976	3 / 7569	2 / 7570	100.00
		1.500	0.251	8.976	3 / 7569	2 / 7570	
Sadoff-Ad26.COV2.S	Viral vector	0.333	0.014	8.179	0 / 21895	1 / 21888	11.35
Voysey-ChAdOx1	Viral vector	0.325	0.066	1.610	2 / 12021	6 / 11724	45.40
Logunov-rAd	Viral vector	0.827	0.161	4.262	5 / 16427	2 / 5435	43.25
		0.488	0.166	1.435	7 / 50343	9 / 39047	
		1.170	0.634	2.162	27 / 85919	21 / 71571	

Heterogeneity: df = 3 (P = 0.23)

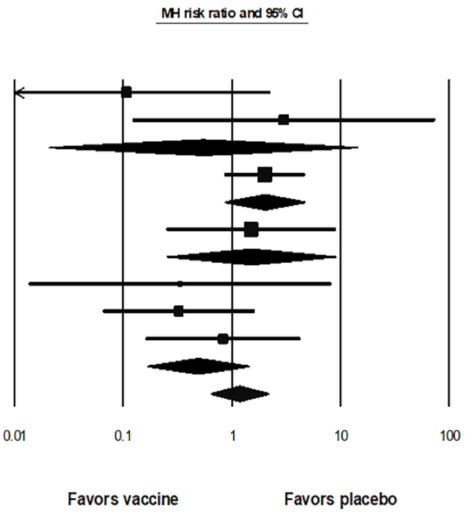

Figure S4 The subgroup analysis to estimate the risk ratio of arterial thromboembolism between the SARS-CoV-2 vaccine and the placebo groups and across vaccine platforms

Study name	Subgroup within study	MH risk ratio	Lower limit	Upper limit	Vaccine	Placebo	Relative weight
Palacios-CoronaVac	Inactivated	3.003	0.122	73.699	1 / 6195	0 / 6201	100.00
		3.003	0.122	73.699	1 / 6195	0 / 6201	
Baden-mRNA1273	mRNA	1.202	0.367	3.936	6 / 15166	5 / 15185	100.00
		1.202	0.367	3.936	6 / 15166	5 / 15185	
Heath-NVX-CoV2373	Protein subunit	0.500	0.045	5.514	1 / 7569	2 / 7570	100.00
		0.500	0.045	5.514	1 / 7569	2 / 7570	
Sadoff-Ad26.COV2.S	Viral vector	3.665	1.023	13.137	11 / 21895	3 / 21888	50.55
Voysey-ChAdOx1	Viral vector	0.975	0.137	6.923	2 / 12021	2 / 11724	30.95
Logunov-rAd	Viral vector	0.331	0.021	5.289	1 / 16427	1 / 5435	18.50
		1.559	0.413	5.889	14 / 50343	6 / 39047	
		1.269	0.568	2.836	22 / 79273	13 / 68003	

Heterogeneity: df = 3 ($P = 0.81$)

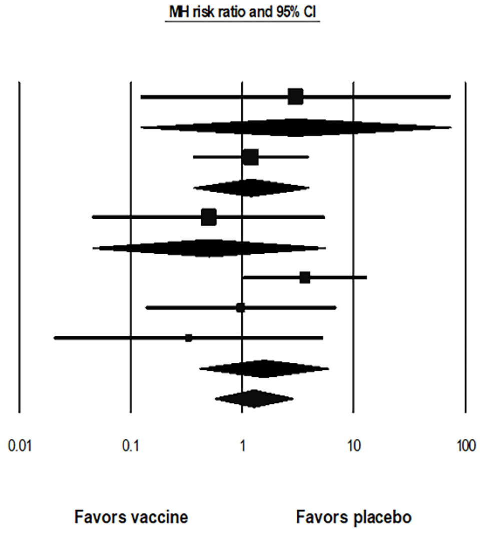

Figure S5 The subgroup analysis to estimate the risk ratio of venous thromboembolism between the SARS-CoV-2 vaccine and the placebo groups and across vaccine platforms

Study name	Subgroup within study	MH risk ratio	Lower limit	Upper limit	Vaccine	Placebo	Relative weight
Tanriover-CoronaVac	Inactivated	1.611	0.066	39.531	1 / 6646	0 / 3568	100.00
		1.611	0.066	39.531	1 / 6646	0 / 3568	
Baden-mRNA1273	mRNA	5.006	0.240	104.269	2 / 15166	0 / 15185	100.00
		5.006	0.240	104.269	2 / 15166	0 / 15185	
Heath-NVX-CoV2373	Protein subunit	1.000	0.063	15.987	1 / 7569	1 / 7570	100.00
		1.000	0.063	15.987	1 / 7569	1 / 7570	
Sadoff-Ad26.COV2.S	Viral vector	2.999	0.122	73.615	1 / 21895	0 / 21888	16.11
Voysey-ChAdOx1	Viral vector	0.731	0.164	3.268	3 / 12021	4 / 11724	67.79
Logunov-rAd	Viral vector	0.110	0.004	2.707	0 / 16427	1 / 5435	16.11
		0.677	0.184	2.485	4 / 50343	5 / 39047	
		0.990	0.350	2.795	8 / 79724	6 / 65370	

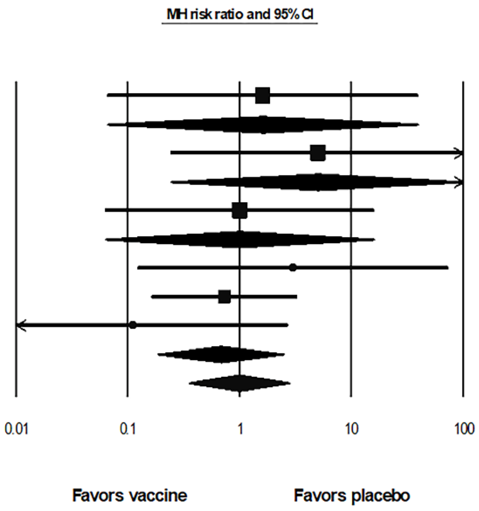

Heterogeneity: df = 3 (P = 0.68)

Figure S6 The subgroup analysis to estimate the risk ratio of hemorrhage between the SARS-CoV-2 vaccine and the placebo groups and across vaccine platforms

Study name	Subgroup within study	MH risk ratio	Lower limit	Upper limit	Vaccine	Placebo	Relative weight
Palacios-CoronaVac	Inactivated	0.334	0.014	8.189	0 / 6195	1 / 6201	100.00
		0.334	0.014	8.189	0 / 6195	1 / 6201	
Baden-mRNA1273	mRNA	1.001	0.063	16.006	1 / 15166	1 / 15185	33.33
Polak-BNT162b2	mRNA	0.999	0.141	7.093	2 / 18860	2 / 18846	66.67
		1.000	0.202	4.954	3 / 34026	3 / 34031	
Sadoff-Ad26.COV2.S	Viral vector	0.333	0.014	8.179	0 / 21895	1 / 21888	50.00
Logunov-rAd	Viral vector	0.110	0.004	2.707	0 / 16427	1 / 5435	50.00
		0.192	0.020	1.843	0 / 38322	2 / 27323	
		0.533	0.159	1.788	3 / 78543	6 / 67555	

Heterogeneity: df = 2 (P = 0.48)

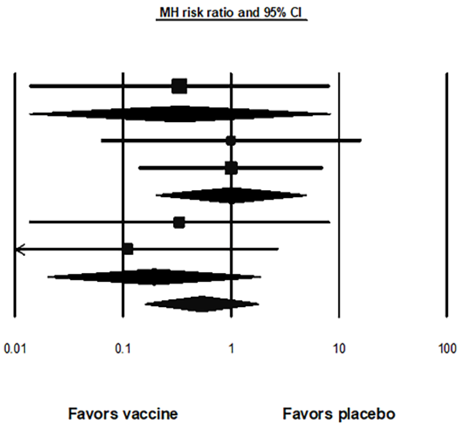

Figure S7 The subgroup analysis to estimate the risk ratio of death related to thromboembolism and hemorrhage between the SARS-CoV-2 vaccine and the placebo groups and across vaccine platforms
